# Automated Systems with Fluorescence Detection for Metal Determination: A Review

**DOI:** 10.3390/molecules29235720

**Published:** 2024-12-04

**Authors:** Arina Skok, Natalia Manousi, Aristidis Anthemidis, Yaroslav Bazel

**Affiliations:** 1 Department of Analytical Chemistry, Institute of Chemistry, Faculty of Science, Pavol Jozef Safarik University in Kosice, Moyzesova 11, 040 01 Kosice, Slovakia; 2 Laboratory of Analytical Chemistry, School of Chemistry, Aristotle University of Thessaloniki, 54124 Thessaloniki, Greece; nmanousi@chem.auth.gr (N.M.); anthemid@chem.auth.gr (A.A.)

**Keywords:** metal determination, automation, flow-injection analysis, sequential injection analysis, fluorescence detection

## Abstract

Industrialization has led to environmental pollution with various hazardous chemicals including pollution with metals. In this regard, the development of highly efficient analytical methods for their determination has received considerable attention to ensure public safety. Currently, scientists are paying more and more attention to the automation of analytical methods, since it permits fast, accurate, and sensitive analysis with minimal exposure of analysts to hazardous substances. This review discusses the automated methods with fluorescent detection developed for metal determination since 2000. It is evident that flow-injection analysis (FIA) with no preconcentration or with solid-phase preconcentration are predominant compared to liquid-phase preconcentration systems. FIA systems are also more widespread than sequential injection analysis (SIA) systems. Moreover, a significant number of works have been devoted to chromatography-based methods. Atomic fluorescence detectors significantly prevail over molecular fluorescence detectors. It must be highlighted that most of the methods result in good figures of merit and performance characteristics, demonstrating their superiority in comparison with manual systems.

## 1. Introduction

Industrialization has led to significant pollution of the environment with various chemicals including toxic metals. The main sources of toxic metal pollution are their mining and handling. Many metals cause serious health problems and have accumulative properties when entering living organisms [1]. On the other hand, some metals are essential for the human body and take part in numerous biological processes. However, these metals may also be toxic at excessive concentrations [2]. Therefore, the development of fast, sensitive, and selective methods for metal determination in environmental samples and in biomaterials is of the utmost importance.

To ensure the compatibility of these samples with analytical techniques and to obtain the desired sensitivity, sample preparation is a mandatory step that can take up to 60% of the time required for analysis [3]. Automatic or on-line sample preparation methods are considered efficient tools, that can provide fast, accurate, and high-precision determination with minimum operator contact with hazardous compounds. Automation also permits us to integrate different analytical processes, such as microextraction with chromatography or capillary electrophoresis. Flow analysis started its development in 1950s, with flow-injection analysis (FIA) that was firstly presented in 1975, and sequential injection analysis (SIA) that appeared in the 1990s [4]. Since that time, researchers have proposed many modernizations and new automated methods to improve sample preparation before determination.

**Figure 1:**
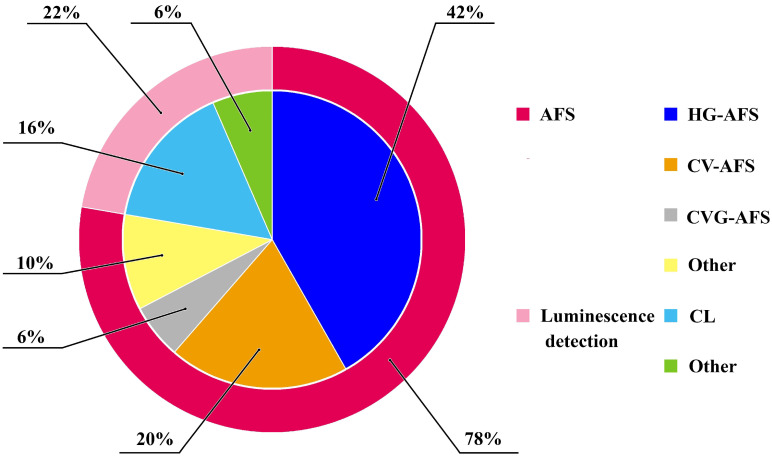
Fluorescence detection methods used in combination with automated procedures.

Fluorescence spectrometry detection techniques are well known for their high sensitivity and selectivity. However, not all the substances have fluorescent properties and this limits the application of these techniques. To overcome this obstacle, chemical reactions that lead to the formation of fluorescence compounds can be carried out. Among the developed automated procedures with fluorescence detection for metal determination, most methods are coupled with atomic fluorescence detection (AFS) (Figure 1). AFS is a cost-efficient and simple technique that permits to obtain high reproducibility and sensitivity with low interferences. It can be employed to determine several elements in complex matrices [5]. This technique can be easily coupled with flow-based and chromatography methods. In around 50% of the examined articles on AFS detection, this technique was combined with hydride generation (HG), cold vapor (CV), or chemical vapor generation (CVG) [6,7].

Since 2000, many automated methods with luminescence detection have been represented in the literature for metal determination. Miro and Hansen presented a review devoted to on-line systems with atomic spectrometry detection for metal determination including AFS [8]. However, they were mainly focused on the technical aspects and innovations of on-line systems. Clough et al. [9] presented a review devoted to atomic spectrometry including AFS updates for metal and other species determination for a 12 month period starting from December 2017. Other reviews have been devoted to the determination of compounds with on-line and automated methods, like arsenic determination using multicommutation flow techniques coupled to hydride generation atomic fluorescence spectrometry (HG-AFS) detection [4], and selenium [10] or antimony [11] determination using AFS detection, including those methods with previous automation.

**Figure 2:**
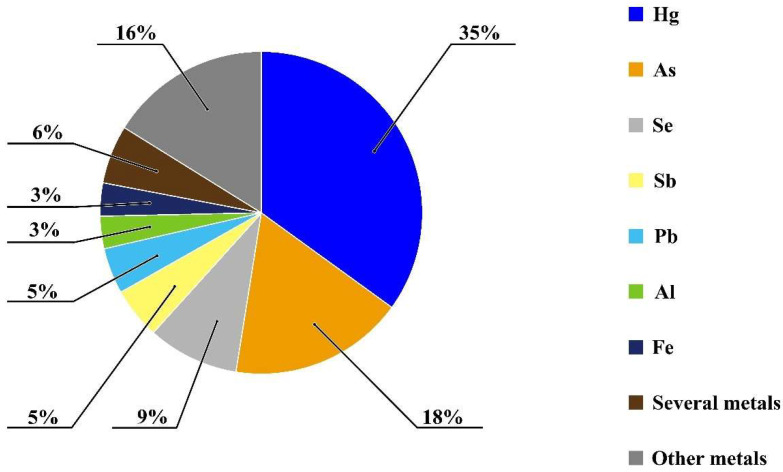
Ratio of articles dedicated to different analytes.

However, to the best of our knowledge, there is no systematic review that describes automated systems with luminescence detection for metal determination. This review is focused on the proposed automated methods with luminescent detection for metal determination reported since 2000. As can be seen from Figure 2, most articles are devoted to Hg, As, and Se detection. The application of FIA and SIA used with or without preconcentration is presented. The combination of flow-based methods with chromatography and other methods (e.g., capillary electrophoresis) is also discussed.

## 2. Continuous-Flow Methods

Continuous-flow analysis is a group of techniques where the sample is injected into a carrier flow and mixed with reagents. Examples of continuous-flow methods are segmented flow analysis, FIA, SIA, and others [12].

Continuous-flow cold-vapor atomic fluorescence spectrometry (CV-AFS) was proposed for Hg(II) and methylmercury determination in water samples [13]. The manifold consisted of two peristaltic pumps, four check valves, a three-way valve, a column packed with silica gel-2-mercaptobenzimidazol, a UV-digestor, a reaction coil, and a gas–liquid separator with a detection system. After washing the system, the sample was pumped through the column for analytes’ preconcentration, while KCN was used for Hg(II) elution. The resulting solution was mixed with SnCl_2_ in the reaction coil, from where it was delivered to the gas–liquid separator. The organic mercury mainly represented by methylmercury was eluted with HCl. Before mixing with SnCl_2_, the second eluate was UV-digested. The LODs were 0.07 and 0.05 ng L^−1^ expressed as Hg for Hg(II) and CH_3_Hg^+^, respectively. The RSDs were 8.8 and 10% for Hg(II) and CH_3_Hg^+^ in river water, respectively. The developed procedure was applied to water samples that did not require any pretreatment except filtration.

Three continuous-flow methods coupled to HG-AFS with different on-line oxidation parts were represented for total arsenic determination in urine in 2010 [14]. For the oxidation, three different procedures were evaluated, namely microwave-assisted heating and UV-photooxidation with or without post-reaction heating. Together with the oxidation parts, the system included two peristaltic pumps, a sample valve, the reaction coil, a gas–liquid separator, and the detection system. The samples were oxidized with K_2_S_2_O_8_ in NaOH and the resulting solution was mixed with HCl and NaBH_4_ before moving to the gas–liquid separator. The detection limits were 9.4, 2.7 and 6.0 µg L^−1^ for microwave-assisted heating and UV-photooxidation with and without post-reaction heating, respectively. The recovery and accuracy were better for UV-photooxidation methods. Moreover, the UV-photooxidation with post-reaction heating also required a shorter analysis time. However, the microwave-assisted oxidation and UV-photooxidation with heating required an additional water cooler, enhancing system complexity.

## 3. Flow-Injection Analysis Systems

### 3.1. FIA Systems with No Preconcentration

FIA is a simple, economic, and convenient automated technique with a high sampling frequency and precision. In its conventional format, FIA is based on the injection of a certain amount of the sample into a carrier or a reagent stream, while in reverse FIA, the reagent is injected into the sample stream. Due to the lower dilution, the sensitivity of reverse FIA is supposed to be higher compared to the conventional approach. The main parts of FIA instrumentation include a pump, an injection valve, the detector, and a signal output device [15,16]. The systems can be equipped with additional parts, such as a gas-diffusion cell with a Teflon membrane [17] and so on.

A FIA-pervaporation-AFS procedure based on microwave-assisted leaching of slurry was proposed for the extractable determination of As in soil samples [18]. The proposed system consisted of a peristaltic pump, an injection valve, a focused microwave and ice water bath, a reaction coil, and the detection system. Each sample was firstly ground and sieved, and slurries were prepared in HCl solution. Slurry mixed with HCl carrier was irradiated with a microwave device, and then it was cooled. After mixing with NaBH_4_, the stream went to the pervaporator and then towards the detection system. Slurry samples were stirred simultaneously with aspiration. The proposed method was approbated on certified reference materials (CRMs) (stream sediment, river sediment, and soil) and different types of soils. The LOD of the developed method was 1 µg L^−1^, the sampling frequency was 4 h^−1^, and the RSD was 4.5%. A potential limitation of the method is that the presence of Sb(III) and Se(IV) can cause interference in the determination.

A reverse FIA procedure with spectrofluorimetric detection was proposed for Al^3+^ determination in tap, commercial, and soft drinking waters [19]. For its quantification, aluminum formed a fluorescence complex with salicylaldehyde picolinoylhydrazone at pH 5.4. The reverse FIA setup consisted of a peristaltic pump, a six-port injection valve, a reaction coil, and a spectrofluorometer. The reagent mixture was injected into the sample flow and the chemical reaction took place in the reaction coil, and the complex was delivered to the spectrofluorometer. Cu^2+^ and Zn^2+^ were masked with thioglicolyc acid, while Fe^2+^ and Fe^3+^ were masked with cyanate. The RSD of this approach was 1.62%. The proposed method was successfully employed for the determination of Al^3+^ in aqueous samples and it resulted in sufficient figures of merit.

A FIA system combined with an ultra-weak chemiluminescence analyzer was proposed for europium determination [20]. In this system, a six-port injection valve connected with one peristaltic pump was responsible for mixing the sample with EDTA, which was further mixed with NaIO_4_ and H_2_O_2_. The obtained solution moved through the second peristaltic pump, into a mixing cell, and was further transferred to the flow cell to measure the analytical signal. From the chemical point of view, the weak chemiluminescence of NaIO_4_–H_2_O_2_ system is proportionally increased with the addition of the Eu^3+^–EDTA complex. The novel system provided a LOD of 6.2 × 10^−8^ M, a sampling frequency of 80 h^−1^, and an RSD of 1.2%. The method was applied for rare earth oxides. As a pretreatment step, mineral powder was dissolved in a HNO_3_ and H_2_O_2_ mixture, evaporated to near-dryness and diluted. A limitation of the method is that terbium greatly interferes with Eu^3+^, so samples with a high presence of it should be avoided.

A simple FIA method with fluorescence detection was proposed for Cr(VI) determination in the wastewater of electroplating baths [21]. This approach was based on the luminescence quenching of ruthenium(II) in the presence of Cr^3+^. Ruthenium(II) and universal pH 4 carrier buffer were mixed in equal proportions with the help of peristaltic pump, then 100 µL of the sample was injected through the injection valve with a sample loop, and the mixture was allowed to reach equilibrium within 1 min in reaction coil prior to its delivering to the detector. The automated method resulted in a two-fold better LOD (i.e., 18 µg L^−1^) and a 10-times-lower required sample volume compared to the manual procedure. A drawback of the method is that Ce^4+^ showed a serious interference effect.

Another fluorescent reaction that can be used for Al^3+^ determination is complexation with morin [22]. To increase the ethanol concentration in the reaction mixture and to enhance the speed of the complexation, FIA was employed. The application of surfactants (i.e., sodium dodecylbenzenesulfonate) was beneficial for the sensitivity of determination. The researchers proposed a normal and a reverse injection system. In reverse FIA, the sample solution was mixed with 0.1 M NaCl, forming the flow with the help of a peristaltic pump, and then morin was injected into the flow with the help of a PTFE simple-injection valve. The obtained mixture moved to the coiled reactor, which was in the thermostat, and then the analytical signal was measured using fluorescence detection. The limit of detection was 2.8 µg L^−1^ in the presence of sodium dodecylbenzenesulfonate and 3.1 µg L^−1^ without it, while the sampling frequency was 90 h^−1^. The RSDs ranged from 2.1 to 2.8%. The proposed method was successfully tested for drinking, river, and underground water analysis.

A flow-injection system was proposed for Al^3+^ determination based on the increase in chemiluminescence properties of a luminol lysozyme system with increased aluminum ion concentration. The FIA system consisted of a peristaltic pump which delivered four streams, a six-way valve with a loop, a mixing tube, a flow cell, and a detector. Pure water was used as a carrier and for system washing before determination. Firstly, luminol was injected using an injection valve into a carrier stream, followed by lysozyme and then the sample solution. The resulting solution was delivered to the flow cell through a mixing tube. The proposed method permitted the detection of aluminum ions in low concentrations with a LOD of 0.1 ng L^−1^. The RSDs ranged from 2.0 to 3.2%. Another important benefit of the system is the high sampling frequency of 100 h^−1^ that was achieved [23].

Zn^2+^ plays an important role in the proper function of the human body, taking part in the synthesis of vital compounds. For this reason, controlling the amounts of electrolytes including Zn^2+^ is important in mineral waters, sport drinks, and some plants. The on-line continuous chelate vapor generation method connected with nondispersive AFS detection was proposed for Zn^2+^ determination in 2017 [24]. This method was based on chelate formation with diethyldithiocarbamate in an acidic medium. The sample and diethyldithiocarbamate solutions were pumped into a three-way connector with a first peristaltic pump, and the resulting mixture was delivered to the gas–liquid separator. Ar was bubbled through the solution to separate gaseous Zn chelate, and the remaining solution was drained with a second peristaltic pump. Zinc diethyldithiocarbamate was delivered to a three-way Y-shaped connector, where it was mixed with H_2_ and delivered to the AFS detector. The proposed system showed a good sensitivity, with a LOD of 0.33 µg L^−1^, and precision, with an RSD of 1.3%. The developed method was tested on plant reference materials that were previously digested with HNO_3_ and HClO_4_. The resulting residue was dissolved in nitric acid and diluted with distilled water with the addition of sodium fluoride which served as a masking agent for metal ions, since sodium diethyldithiocarbamate is a strong chelating agent towards many different metals.

A FIA system could also be applied for studying the effect of masking agents on particular processes under the matrix with interferents, such as PbH_4_ formation in HG-AFS [25]. Other analytical issues, like the influence of the species effect on the trueness of the analytical results [26], or the characterization of some chemical processes [27], were also studied using automated systems.

Table 1 describes other FIA procedures used for metal determination with fluorescence detection.

### 3.2. FIA Systems for Solid-Phase Preconcentration

Solid-phase extraction (SPE) permits us to effectively preconcentrate analytes and/or separate them from possible matrix interferences. In its automated alternative, SPE is usually achieved by the incorporation of packed or disk-phase-based columns into the flow system. The literature presents a huge selection of all kinds of sorbents for various purposes. The drawbacks of SPE are the possible creation of back pressure and analyte carryover [8]. As an alternative to column SPE systems, knotted reactors are also used for solid-phase preconcentration. They mainly consist of twinned knotted PTFE tubes on which uncharged analytes are retained with molecular sorption or hydrophobic forces. Reactors change flow direction with the help of knots, producing centrifugal force that delivers the analyte towards the inner walls, where it is retained. They have a low flow resistance, and they are simple in application and production. However, compared to resin-packed columns, knotted reactors have a weaker retention efficiency [55].

An on-line flow-injection system coupled with an HG-AFS detector was proposed for germanium determination [56]. The determination procedure was based on its coprecipitation with nickel hydroxide and further dissolution with 20% (*v*/*v*) H_3_PO_4_ solution, then its reaction with NaBH_4_ and fine transference to the detector. A FIA multifunction solution autohandling system was employed and it mainly consisted of eight channel rotary injection valves, two peristaltic pumps, a reacting coil, and a precipitate collector. Sufficient sensitivity and precision was attained, with a LOD of 0.11 µg L^−1^ and an RSD of 5.6%, while the proposed method was tested on two standard reference materials.

A FIA-HG-AFS procedure for cadmium determination in seawater was proposed by Wu et al. [57]. In seawater, cadmium may be present due to industrial pollution, and it is highly toxic even in trace concentrations. In this procedure, cadmium determination was based on its complexation with 1-phenyl-3-methyl-4-benzoylpyrazol-5-one. The obtained complex was retained in the knotted reactor and HCl was used for complex elution. KBH_4_ mixed with HCl was applied to define the baseline signal. The obtained sensitivity of the method was good (i.e., LOD: 3.2 ng L^−1^), and the sampling frequency was satisfactory (i.e., 24 h^−1^). The developed procedure was successfully tested on CRM and seawater samples, demonstrating a good performance.

The same year, a FIA-HG-AFS system with on-line preconcentration was proposed for lead determination [58]. In the FIA system, a knotted reactor was attached to the injection valve for the preconcentration. Initially, an ammonium buffer and the sample were pumped through the knotted reactor and Pb(OH)_2_ was collected. Air segments were used to displace the remaining liquid from the reactor. At the same time, KBH_4_ and HCl were directed to the gas–liquid separator. For quantification, HCl passed through the knotted reactor, dissolving the precipitate and delivering it to the gas–liquid separator. The sample throughput, enhancement factor, and detection limit were 30 h^−1^, 16, and 16 ng L^−1^, respectively. Thus, a good method performance was obtained. The RSD was 3.4%. The proposed procedure was applied for water, wine, and rice analysis.

Bismuth was determined in water and urine samples with a FIA-HG-AFS method developed by Wu et al. [59]. A column packed with nylon fiber was used for preconcentration of a bismuth complex with Bismuthiol I. The elution of the obtained complex was performed using a HCl solution and the resulting solution and KBH_4_ moved to the gas–liquid separator and then to the detector. A sampling frequency of 24 h^−1^ was achieved, together with a good method sensitivity (i.e., LOD: 2.8 ng L^−1^) and precision (i.e., RSD was 4.4%). The method was used for CRM (tea and hair), water, and urine sample analysis.

On-line preconcentration of zinc combined with HG-AFS detection was proposed for its monitoring in water and sport drinks [60]. The proposed system consisted of a peristaltic pump, a six-way valve that incorporated the preconcentration minicolumn, and a detector. Polyurethane foam was used as a sorbent packing material for the column, while HCl was employed for the elution step. Zinc hydride was generated using NaBH_4_. The detection limit was calculated as 0.03 µg L^−1^, and the resulted sampling frequency was 51 h^−1^. A benefit of the proposed system is the high enrichment factor that was achieved for zinc (i.e., 88.92).

An on-line chemiluminescence system was proposed for copper determination in wastewater [61]. Cu^2+^-imprinted cross-linked chitosan resin was applied as a column packing material. The flow-injection analyzer consisted of two pumps, a switch valve, a microcolumn, and a detection system. The method was based on the luminol–hydrogen peroxide chemiluminescence system. The column was placed in front of the window of the photomultiplier tube of the detector. The obtained LOD was 32 nmol L^−1^, and the method was linear within a range of two orders of magnitude. Moreover, the RSDs ranged from 4.7 to 5.3%. A significant advantage of the proposed method is that it improved the shortcomings of the chemiluminescence method’s poor selectivity. Moreover, the herein-used microcolumn was found to be reusable 200 times, serving as an additional benefit.

A FIA system with chemiluminescent detection was proposed for V(V) determination in natural water samples [62]. V(V) is toxic for animals and humans, so the control of its amount in natural and drinking water is of great importance. The herein-used FIA system was based on the property of V(V) to increase the chemiluminescence intensity of a luminol–periodate mixture. Thus, the analytical signal was calculated as the difference between the chemiluminescence signal in the presence and absence of V(V). In this approach, a LOD of 60 ng L^−1^ was found, together with a sampling frequency of 120 h^−1^ and RSDs between 1.45 and 3.9%. Good performance characteristics were obtained, and the developed method was successfully used to determine vanadium in natural waters as confirmed by ion chromatography.

The total vanadium content of seawater samples has been determined with a FIA system combined with chemiluminescent detection [63]. The sample was injected into a sulfuric acid carrier stream, and then it moved through an amalgamated Zn column. This caused the reduction of all vanadium forms to V(III). The stream with vanadium was merged into the combined stream of potassium permanganate and formaldehyde. The resulting mixture passed through a flow cell, where the analytical signal was measured with a photomultiplier tube. The appearance of chemiluminescence is caused by V(III) or V(II) reacting with potassium permanganate in the presence of formaldehyde in an acidic medium. The LOD was 40 ng L^−1^ and the sampling frequency was 100 h^−1^. The RSDs ranged from 1.8 to 3.1%. The proposed method was tested on seawater and CRM, and it exhibited good performance characteristics. A limitation of this system is that Fe^2+^, Mn^2+^, Sn^2+^, SO_3_^2–^, and I^–^ can cause interfering effects. However, anion interference can be eliminated by using an anion-exchange column.

More than one column can be used simultaneously in preconcentration systems. An example of such FIA-HG-AFS systems was represented for lead determination in 2009 [64]. The multifunction solution autohandling system consisted of an injection valve, three peristaltic pumps, two ion-exchange microcolumns with D401 chelating resin and the detector. The sample solution was pumped through the columns simultaneously and the retained Pb^2+^ was subsequently eluted by HCl. NaBH_4_ was used for volatile hydride formation. The utilization of the two columns permitted the researchers to achieve a higher analytical signal, resulting in a LOD of 3.1 ng L^−1^, an RSD of 3.78%, and a sampling frequency 15 h^−1^.

Malejko et al. [65] proposed a flow-injection chemiluminescence method for the determination of Pt(IV) based on the luminescence quenching of lucigenin [65]. The FIA system consisted of two peristaltic pumps, an injection valve, a column packed with algae *C. vulgaris* immobilized on Cellex-T resin, a reaction coil, and the flow cell. In this case, the analytical signal was calculated as the difference between the background emission (lucigenin without Pt(IV)) and the reagents’ emission in the presence of platinum. An adequate sampling frequency (i.e., 4 h^−1^) and sensitivity (i.e., LOD: 0.1 µg L^−1^) were achieved. The RSDs were 2.0 and 2.9%. The proposed method was successfully employed for river water and dust sample analysis.

An SPE-based FIA-HG-AFS procedure using an ion-imprinted polymer column has been proposed for Bi(III) determination [66]. The sorbent was synthesized from 2-(5-bromo-2-pyridylazo)-5-diethylaminophenol, ethylene glycol dimethacrylate, cross-linking reagent, and methacrylic acid. HCl was employed as an eluent and NaBH_4_ for hydride generation. The obtained detection limit was 26 ng L^−1^, the RSD was 3.7%, and the sampling frequency was 13.3 h^−1^, and the proposed method was successfully used for seawater analysis. Table 2 describes other FIA procedures with SPE reported in the literature between 2000 and 2024.

### 3.3. FIA Systems for Liquid-Phase Microextraction

Automation permits us to decrease sample and reagent consumption, reduce the amount of waste, avoid contamination, and also simplify analysis in the case of liquid-phase microextraction [94]. Thus, various FIA systems have been developed for the automation of liquid-phase microextraction, although these techniques are reported less often compared to SPE.

On-line extraction combined with HG-AFS has been applied for mercury determination [95]. The system consisted of two peristaltic pumps, extraction, mixing, and reaction coils, an injection valve, a gravity separator, a gas–liquid separator, and the detector. The analyte was extracted with tributyl phosphate inside the extraction coil. Then, the extraction solution was delivered to the gravity separator and the extractant was mixed with acetic acid and NaBH_4_ in *N*,*N*-dimethylformamide. The LOD was 20 ng L^−1^, showing a good sensitivity. The proposed method was tested on CRMs (soils and fly ash).

Arsenic in soils was determined with flow-injection on-line sequential extraction coupled with HG-AFS [96]. For the sample preparation, a FIA-3100 model equipped with two peristaltic pumps, a standard rotary injection valve, a microcolumn, a standard solution loop, and a detection system including a gas–liquid separator was used. The microcolumn was packed with soil samples that were sequentially extracted with water, KOH, and HCl. The extractant was pumped through the microcolumn and then mixed with K_2_S_2_O_8_ in KOH. As(III) was oxidized to As(V). Next, the solution of the loop was pumped using HCl and mixed with extractant and KBH_4_ and delivered towards the detection system. The sampling frequency was 6 h^−1^ and the method was validated with two soil CRMs, showing a good performance.

On-line electrokinetic extraction with electrochemical HG-AFS detection was proposed for inorganic As determination in water [97]. The extraction and the electrochemical hydride generation was performed in an H-type integrated cell. Two peristaltic pumps were used to deliver the solutions. The system also included two gas–liquid separators and an AFS detector. As(III) was oxidized to As(V) with H_2_O_2_ for total inorganic As determination, and good sensitivity and precision were achieved, since the LOD for As(V) was 0.020 µg L^−1^ and the RSDs were 2.3–3.5%.

On-line extraction with reversed micellar-mediated chemiluminescence detection was proposed for Sb(III) and Sb(V) determination [98]. The flow-injection analyzer comprised a 16-port injection valve, three plunger pumps, an extraction coil, a phase separator, and the detector. The sample solution was mixed with rhodamine B before conducting the analysis. The sample and toluene streams were mixed and delivered to the extraction coil and then to the phase separator. Accordingly, the toluene containing the extracted Sb chloride complex phase passed through a Teflon membrane and moved to one of the inlets of the detection system. Then, the reversed micellar reagent solution containing the Ce(IV) oxidant, 1-hexanol-cyclohexane, and cetyltrimethylammonium chloride was delivered with the carrier stream to the flow cell and mixed with the toluene. Sb(III) was oxidized to Sb(V). After this procedure, the excess of Ce(IV) was reduced with hydroxylamine and the analytical signal was measured. For Sb(V) determination without Sb(III), Ce(IV) was not added and the LOD was 0.35 µmol dm^−3^ for Sb(V). The method was tested on water and copper electrolyte liquid samples after pretreatment. The same research group proposed a similar system for Au(III) and Ga(III) determination the same year [99]. Au(III) was extracted with 2 M HCl solution, while for Ga(III) a 5.0 M HCl solution with 2.5 M LiCl was used. The LODs were 0.4 μM and 0.6 μM for Au(III) and Ga(III), respectively.

### 3.4. Multisyringe FIA Systems

In addition to the other important FIA components, multisyringe FIA systems include a multisyringe burette with four syringes that move simultaneously. For reducing reagents and samples consumption, each syringe is connected to a three-way solenoid valve. Using the valve, any solutions that are not required are sent back to the stock solution [4].

The total inorganic arsenic level was determined using a time-based multisyringe FIA-HG-AFS in 2002 [100]. The system included a multisyringe burette with four syringes, of which three were used, five solenoid valves, an auto-sampler, a glass gas–liquid separation cell, reaction and sample coils, a permapure dryer, and the detector. The sample, KI, and NaBH_4_ were injected into the system and mixed before going to the gas–liquid separator. KI with ascorbic acid (for avoiding I^-^ oxidation) was used to reduce As(V) to As(III) and subsequently the gaseous arsenic moved to the dryer and the detector. The LOD of the system was 0.07 µg L^−1^ with a high sampling frequency of 108 h^−1^.

A time-based multisyringe FIA with spectrofluorimetric detection was also proposed for aluminum detection in drinking water in the same year [101]. The proposed system combined the advantages of both FIA and SIA systems. The system consisted of a multisyringe burette with four syringes and solenoid connection valves connected to them, a sample coil, a reaction coil, a solenoid valve, and a spectrofluorimeter. In the proposed method, only three syringes were used: the first syringe for sample delivering, the second for carrier delivery, and the last for reagent mixture delivery. Luminescence was observed after Al^3+^’s reaction with 8-hydroxyquinoline-5-sulphonic acid in a micellar medium created by hexadecyltrimethylammonium chloride at a pH of 6. The LOD was 0.5 µg L^−1^.

Inorganic arsenic was determined using a multisyringe flow-injection system with HG-AFS detection [102]. The system contained a multisyringe burette with four syringes, six three-way solenoid commutation valves, sample and cleaning coils, a cross-shaped (five-way) connector, a column, a gas–liquid separator followed by a permapure membrane dryer and the detector. The syringes were responsible for delivering HCl; water, which was used for column cleaning together with NaBH_4_ introduced through one of the valves; NaBH_4_; and the sample. The column was packed with anion-exchange resin (Amberlite IRA-410) and the arsenic ions that were preconcentrated on the column were further eluted with HCl. The system showed a LOD of 30 ng L^−1^, an RSD of 4.8%, and a sampling frequency of 10 h^−1^.

A multisyringe FIA-HG-AFS for dimethylarsinic, inorganic arsenic, and total arsenic determination was proposed in 2012 [103]. The system consisted of a multisyringe burette with four syringes, six solenoid valves, an auto-sampler, holding and reaction coils, a photoreactor for sample irradiation with a UV lamp, gas–liquid separator, a permapure dryer, and the detector. Depending on the pretreatment procedure for CRM and reagents used, different forms of As were determined. When the sample was extracted with water and measured directly with the addition of HCl, NaBH_4_ for hydride generation, KI, and C_6_H_6_O, the total inorganic arsenic level was determined. When the extractant was protoxidized with the addition of K_2_S_2_O_8_, HCl, and NaBH_4_, the levels of inorganic arsenic and dimethylarsinic were determined simultaneously. Finally, after sample digestion with HNO_3_ and H_2_O_2_, the total As level was directly measured in the system. Dimethylarsinic was determined with a LOD of 0.09 µg L^−1^ and a sampling frequency of 24 h^−1^, while for inorganic arsenic the LOD and sampling frequency were 0.47 µg L^−1^ and 28 h^−1^, respectively.

Sb(III), Sb(V), and trimethylantimony were determined in water samples using multisyringe FIA-HG-AFS [104]. The proposed FIA system included a burette with syringes filled with HCl, NaBH_4_, KI with ascorbic acid and a carrier for sample delivering, six solenoid valves, a DOWEX^®^ 50 WX8 minicolumn, two reaction coils, a gas–liquid separator, a permapure dryer, and the detector. Firstly, Sb(III) was determined. The sample passed through the column, where trimethylantimony was retained. Then, the sample solution was mixed with HCl and NaBH_4_ in the reaction coil, before their delivery in the gas–liquid separator. A similar procedure was followed for inorganic Sb determination (Sb(III) and Sb(V)). The only additional step required was the reduction of Sb(V) to Sb(III) with KI in the first reaction coil before mixing with HCl and NaBH_4_. The total antimony was determined by allowing bypass to the minicolumn. The LODs were 0.03 and 0.13 µg L^−1^ for inorganic Sb forms and trimethylantimony, respectively, with a 30 h^−1^ sampling frequency for all species. The RSDs were 2.8 and 3.8% for inorganic Sb and trimethylantimony, respectively. The real samples required filtration, acidification, and 8-hydroxyquinoline addition before conducting analyses.

Other multisyringe FIA procedures are described in Table 3.

## 4. Sequential Injection Analysis Systems

### 4.1. SIA Systems with No Preconcentration

Until now, different automated SIA systems combined with fluorescence detection have been developed for metal determination. The main components of SIA systems include a selection valve, a pump, a channel, and the detector. Every valve port is responsible for a specific purpose, which provides good versatility. The SIA procedure is based on the sequential injection of different components, like the reagent, sample, or washing solution. It provides a low reagent and sample consumption with a small amount of waste [15]. SIA systems can also be more multiparametric and robust than FIA-based ones. Both methods are simple in operation; however, when researchers need to change the procedure of analysis, SIA needs only a change in program, while FIA often requires a change in hardware.

A SIA-HG-AFS was proposed for total inorganic arsenic determination in 2000 by Semenova et al. [111]. The system was fabricated from a burette with a syringe, an 8-channel selection valve, an auto-sampler, a glass gas–liquid separation cell, and a detector. Initially, the sample together with a blank containing HCl, KI, and ascorbic acid and NaBH_4_ (reducing agent) was injected into the system. Then, with reversed flow, the mixture moved to the gas–liquid separator. The resulting gaseous analyte was delivered to the detector with Ar, and KI was used for converting As(V) to As(III). The detection limit was 0.67 µg L^−1^, the RSD was 1.8%, and the sampling frequency was 33 h^−1^.

SIA and batch procedures with fluorometric detection have been proposed for labile forms of Al^3+^ determination in tap and drinking waters [112]. The aluminum detection was based on its complexation with 8-hydroxyquinoline-5-sulfonic acid at pH 4 acetate buffer. The interference from Fe^3+^ was eliminated by adding hydroxylamine chlorhydrate as a reducing agent. The SIA system consisted of eight-port selection valves to which a spectrofluorometer and a reaction coil with an automatic burette were connected. Initially, the reagent and sample were delivered to the reaction coil and then the mixture was injected into the detector. Compared with the batch procedure, a higher limit of detection and a lower sampling frequency was found.

A SIA system consisting of a peristaltic pump, a selection valve, holding and reaction coils, and a fluorometric detector was represented for Sn determination in the juices of canned fruits [113]. DMSO, the sample solution, and 8-hydroxyquinoline-5-sulfonic acid were sequentially aspirated and propelled using acetate buffer carrier. A benefit of this approach is the high sampling frequency (i.e., 70 h^−1^) that was achieved. However, the obtained detection limit was relatively high (i.e., 380 µg L^−1^), reducing the overall method sensitivity.

SIA-CV-AFS was used for mercury determination in sediments by Leng et al. [114]. In this case, the sample was delivered with a carrier solution into a mixture cell, where it was mixed with NaBH_4_. The resulting vapor was transferred to the gas–liquid separator. A LOD of 0.5 ng g^−1^ and an RSD of 3.7% were achieved, and the method was tested on CRM and sediments, showing a good performance.

A SIA-HG-CL protocol was proposed for selenium determination in 2016 [115]. The system consisted of a syringe pump with a 10 mL syringe connected to an eight-port switching valve, three three-way solenoid valves, hot and cold reactors, and the detector. Firstly, the sample and KMnO_4_ in H_2_SO_4_ were pumped to the hot reactor through the cold one, where organic Se was decomposed. The resulting mixture was transferred back to the cold reactor. After cooling, it was mixed with NaBH_4_ in order to remove As. After KBr addition, the solution was transferred to the hot reactor, where Se(VI) reduction to Se(VI) was performed. Finally, the mixture was delivered to the cold reactor, where NaBH_4_ was added to obtain volatile H_2_Se. During the As and Se hydride generation steps, the gaseous phase was sent to the CL with O_3_. The LOD was calculated as 8 µg L^−1^. Thermal-power-plant wastewaters were tested as a real sample without any pretreatment. A drawback of this approach is the multiple steps that are required and the fact that it is time-consuming.

Inorganic Hg and total Hg were determined with an on-line digestion procedure using CV-AFS detection [116]. The system comprised a syringe pump, a selection valve, a holding coil, a digestion vial with a magnetic stirring bar, a peristaltic pump, a reduction coil, and the detection system. The sample was mixed with HCl and KMnO_4_ (for total Hg determination, Na_2_S was also added) and delivered to the digestion vial. The resulting mixture was mixed with HCl, ascorbic acid, and NaBH_4_ and delivered to the gas–liquid separator. The detection limit was 3 ng L^−1^ and the sampling frequency was 10 h^−1^. The RSDs were from 1.1 to 3.1%. Finally, the method was tested for its efficiency in a water sample.

### 4.2. SIA Systems for Solid-Phase Preconcentration

A SIA-HG-AFS procedure was developed for inorganic selenium determination in 2009 [117]. The system comprised a syringe pump, two holding coils, a six-port selection valve, a microcolumn, a gas–liquid separator, and the detector. Se(VI) was pre-reduced to Se(VI) by heating with hydrochloric acid for total inorganic selenium determination. The SPE microcolumn was packed with La(OH)_3_-coated cellulose fiber, and Se(IV) was firstly precipitated on a column. For the elution, NaBH_4_ in NaOH was used and the eluate was delivered with HCl to the gas–liquid separator and detector. The detection limit and the sampling frequency were calculated as 9 ng L^−1^ and 24 h^−1^, respectively. The RSD of the proposed approach was 1.7%. Together with the good sensitivity, a good method performance was proved by the analysis of CRM (rice), hair, and water samples.

Chen et al. [118] proposed a sequential-injection on-line mercury speciation and preconcentration system as a front-end to CV-AFS. Cellulose fiber functionalized with l-cysteine was used as a column packing material that was used for the preconcentration of the mercury species on the column. As eluent, l-cysteine with HNO_3_ was used and the resulting solution was mixed with NaBH_4_ and delivered to a gas–liquid separator prior to mercury determination. The LODs were 1 ng L^−1^ for inorganic Hg and 3 ng L^−1^ for CH_3_Hg with a sampling frequency of 12 h^−1^, demonstrating a good overall method performance. The RSDs were 1.5 and 2.6% for Hg and CH_3_Hg, respectively. The applicability of the developed method was demonstrated for CRM analysis (city waste incineration ash), cosmetic samples, and seaweed.

An on-line SPE-HG-AFS based on SIA was proposed for inorganic As determination in 2017 [119]. The on-line system consisted of a changeover valve, two injection pumps, a sample loop, a column, a reacting mixture, a gas–liquid separator, and a detection system. The column was packed with polystyrene resin that was able to retain As(III), which was then eluted using water and delivered to the vapor generator. After mixing with KOH and KBH_4_, the resulting solution was delivered to gas–liquid separator. The AsH_3_ signal was measured with AFS, providing a LOD of 3 μg kg^−1^ LOD. The RSDs were 2.2 and 3.1% for Hg and CH_3_Hg, respectively. The proposed method was successfully used for the analysis of algae samples.

### 4.3. SIA Systems for Liquid-Phase Preconcentration

A SIA-based protocol for the on-line multichannel ultrasonic extraction of arsenic combined with HG-AFS determination was developed in 2013 [120]. The proposed instrumentation consisted of a peristaltic pump, two six-port multi-position sequential injection valves, an ultrasonic bath, a three-port valve, a pre-reduction reactor in thermostatic water bath, and the detection system. Firstly, the sample and HCl were delivered into tubes that were immersed in an ultrasonic bath using the first sequential injection valve. After this, the resulting solution was delivered to a pre-reduction reactor, where it was mixed with a thiourea ascorbic acid solution and As(V) was pre-reduced to As(III). Then, the solution was mixed with KBH_4_ and delivered to the gas–liquid separator and then to the detector. The developed method was applied for As determination in CRM soil samples that were first ground and sieved, followed by diluted with deionized water to form slurries. Satisfactory sensitivity was achieved since the LODs for different soil CRMs varied from 30 to 70 ng g^−1^. However, the complexity of the proposed system is increased since it also requires the utilization of an ultrasonic bath.

## 5. Combination of Flow-Based Methods with Chromatography and Other Methods

Different chromatography techniques (i.e., liquid and gas chromatography) are often coupled on-line with flow-based techniques to achieve better analytical parameters. As an example, Camurati et al. [121] developed an analytical protocol for the on-line speciation analysis of arsenic compounds (i.e., As(III), As(V), monomethylarsonic acid, dimethylarsinic acid, and arsenosugars) in commercial edible seaweed by high-performance liquid chromatography–ultraviolet detection (HPLC–UV) combined with thermo-oxidation-HG-AFS. The proposed system consisted of an HPLC pump, a syringe injection valve with loop, an anion-exchange column, a UV system, a heating system, and a detection system. After passing through the HPLC column, the eluate was UV-thermo-oxidized with K_2_S_2_S_8_ in NaOH and cooled in an ice bath, then mixed with NaBH_4_ in NaOH and HCl streams prior to gas–liquid separation and detection. The achieved LODs ranged from 2.6 to 17 ng g^−1^, providing sufficient sensitivity. However, the complexity of the analytical technique is increased, reducing its potential applicability in quality control laboratories.

Capillary electrophoresis (CE) could be considered as alterative technique towards chromatography in terms of separation. It is simple, requires a small amount of sample and reagents, and provides efficient separation [7]. Different on-line systems for metal determination have been also coupled with CE. Table 4 summarizes the on-line systems using chromatographic and electrophoretic separation techniques.

## 6. Conclusions

Due to human activity, pollution with metals has become a great problem in recent years. On the other hand, some metals play an essential role in the proper functioning of the human body. Automated techniques became a recent trend in analytical chemistry because they can provide fast, easy, safe, accurate, and high-precision analysis. Interest towards automated techniques has only increased over the years. This work describes the automated methods with luminescence detection proposed for metal determination since 2000. Among the proposed methods, SPE-based FIA methods prevailed over SIA and liquid-phase-extraction approaches. The researchers also paid significant attention to chromatography-based on-line methodologies. Around one-third of all articles were devoted to Hg analysis. Multiple articles were represented for As and Se determination. Water was the most common matrix for real sample analysis, while a considerable number of methods were developed for the analysis of biomaterials and soil samples. Proper detector selection is also an important part of analytical method development. AFS detection combined with hydride generation was the most common detection technique. Several methods were also proposed in combination with molecular luminescence detection; most of them are based on luminescence provoked by chemical reactions. Human activity has led to serious environmental problems. Due to the global warming and environmental pollution, humanity is trying to switch to more environmentally friendly technologies to solve this problem. This effort is reflected in analytical chemistry as the principles of green analytical chemistry. Miniaturization and automation are important parts of these principles that correspond to the development of new automated FIA and SIA procedures that do not require a high consumption of solvents and produce a small amount of waste. Thus, more and more attention are being dedicated to automation. The other part of this process is replacing hazardous compounds with more ecofriendly ones. It is also necessary to consider the matrix effect on analyte determination. Most water matrices do not require any additional sample pretreatment except filtration. Other sample matrices are more complex and may require digestion or previous extraction in the case of solid matrices. Soil samples may be converted into slurries before analysis. Several procedures incorporate sample pretreatment in the automated determination of metals. In the future, we assume there will be undying interest in the development of new environmentally friendly automated methods for metal determination. New automated approaches would be represented with the increasing interest to the flow-based methods. Finally, AFS detection is also expected to prevail in molecular luminescence detection.

## Figures and Tables

**Table 1:** FIA procedures for metal determination with fluorescence detection.

Analyte	Analytical Technique ^1^	Sample(s)	Sample Pretreatment	LOD (µg L^−1^)	Sampling Frequency (h^−1^)	RSD (%)	Ref.
As(III)/As(V)	HG-AFS	Soil	Sequential extraction with KH_2_PO_4_/HCl/NaOH and digestion	0.11/0.07	20	1.43	[28]
Total As	HG-AFS	CRM (Lichen, Lagarosiphon major, Platihypnidium riparoides, river sediment), leaves from Acacia dealbata	Leaves drying and grinding, microwave digestion with HF, HNO_3_, ultrasonic extraction with HCl	0.03–0.15 µg g^−1^	NA ^2^	3.1	[29]
As(III)	Gas-phase CL	NA	NA	0.6	450	NA	[17]
As(V)	CL	Water	Chelex 100 column (removing interference), ammonium molybdate, luminol	0.15	120	0.8–2.5	[30]
As(III)	HG-AFS	CRM, water	Dilution with Tris buffer	0.027	NA	˂3.0	[31]
As(V)				0.036			
As(III)	HG-AFS	water	Filtration, HCl addition	0.02	10	≤2.09	[32]
Total inorganic As			Filtration, thiourea, ascorbic acid, HCl addition				
Pb(II), Hg(II), Cu(II)	Bioluminescence	water	NA	NA	10^−4^ M, 10^−5^ M, 10^−4^ M	0.7	[33]
Co(II)	CL	Water	Filtration, centrifugation, dilution, previous mixing with lucigenin	67 pM	NA	0.61–1.18	[34]
Fe(II)	CL	Water	Seawater collection on the ship, in-line filtration, analyses on board	77 pM	33	NA	[35]
Fe(II)	CL droplet detector	Artificial seawater	NA	7.16 nmol dm^−3^	198	0.82–3.74	[36]
Fe(III)	Luminescence	Industrial effluents	Filtration, Fe(II) oxidation with H_2_O_2_	3.4	120	0.6–1.6	[37]
CH_3_Hg+, Hg(II)	CV-AFS	CRM (dogfish muscle, lobster hepatopancreas)	Slurry formation with Triton XT114, HCl, H_2_SO_4_, HNO_3_, H_2_O_2_, K_2_Cr_2_O_7_	0.07	NA	6.8	[38]
Inorganic Hg	CV-AFS	Blood, red blood cells, plasma, hair, urine	Solubilization: l-cysteine, NaOH addition, NaCl addition (hair)	0.01–0.09	NA	NA	[39]
Total Hg							
Hg	AFS	Cigarette smoke	Smoke collection into KMnO_4_ in H_2_SO_4_ solution	0.0038	NA	2.7	[40]
CH_3_Hg, Hg(II)	CE-VSG-AFS	Water	NA	200, 100	60	≤4.2	[41]
Total Hg	CV-AFS	Catfish muscle tissues	Microwave-assisted extraction with HNO_3_, H_2_O_2_	4 ng g^−1^	NA	˂9	[42]
Inorganic Hg			Microwave-assisted extraction with tetramethylammonium hydroxide	26 ng g^−1^			
Total Hg	CV-AFS	Water	Filtration	0.000016	13	NA	[43]
Hg	CV-AFS	Serum blood	Slurry sampling in the presence of aqua regia and antifoam	0.025	NA	3.9	[44]
Inorganic Hg	CV-AFS	CRM (fish tissue)	Extraction with tetramethylammonium hydroxide	4.3 ng g^−1^	NA	1.3	[45]
Total Hg				3.7 ng g^−1^			
Hg(II)	CL	Water	Filtration, immunoassay Based on resin beads preparation	0.015	NA	4.37	[46]
Hg(II)	CL	Water	Previous mixing with nanoparticles	8.6 nM	NA	NA	[47]
Total Hg	CV-AFS	Hair	Digestion with HNO_3_ and H_2_O_2_	4 µg kg^−1^	60	3.2–6.0	[48]
Hg(II) (thiomersal)	Luminescence	Vaccine	Photodegradation of thiomersal to Hg^2+^ with H_2_O_2_	NA	90	NA	[49]
K^+^	G-Quadruplex AGRO100, hemin DNAzyme-enhanced CL	Serum	Digestion with HNO_3_, HClO_4_	0.69 μmol L^−1^	60	1.72	[50]
Mn(II)	CL	Water	Incorporated 8-HQ column for interference removal	0.1	90	1.0–3.4	[51]
Pb	HG-AFS	CRMs (water, lyophilized muscle tissue), water, blood, plasma, serum	KI as a masking agent for water; for other samples, digestion with HNO_3_	0.03	70	1.1	[52]
Sb	HG-AFS	Brass	Digestion with HNO_3_, precipitation with NaOH, supernatant analysis	1.89	NA	2.3–3.5	[53]
Sb	HG and cryotrapping gas-phase CL	Water, soil	Filtration (water); digestion with HNO_3_, HCl, HF (soil); acidification, l-cysteine addition (interference elimination)	0.18	15	3.56	[54]
Total Se	HG-AFS	Water	NA	NA	NA	NA	[26]

^1^ CL: Chemiluminescence detection; CE-VSG-AFS: Capillary electrophoresis volatile species generation atomic fluorescence spectrometry; CRM: Certified reference material; CV-AFS: Cold-vapor atomic fluorescence spectrometry; HG-AFS: Hydride generation atomic fluorescence spectrometry. ^2^ NA: Not available.

**Table 2:** FIA procedures with column/knotted reactor separation for metal determination with fluorescence detection.

Analyte	Analytical Technique ^1^	Sample	Sample Pretreatment	Reagents	Sorbent	LOD (µg L^−1^)	Enrichment Factor	Sampling Frequency (h^−1^)	RSD (%)	Ref.
Column										
Total As	HG-AFS	Food	Digestion with HNO_3_ and HClO_4_, l-cysteine addition	APDC, HCl (eluent), KBH_4_	Cigarette filter	3.5–9.9 ng g^−1^	26.5	11.6	1.1–2.2	[67]
As(III)	HG-AFS	High purity Sb_2_O_3_	Decomposition with HCl	HCl, KBH_4_ in NaOH	Amberlite XAD-16	0.26 µg g^−1^	NA ^2^	NA	6.2	[68]
As(III)	Double-channel HG-AFS	Water, CRM	Filtration	APDC, KBH4, HNO_3_	Single-walled carbon nanotubes	0.0038	25.4	NA	4.2	[69]
Sb(III)						0.0021	24.6		4.8	
As(V) (+As(III))			Filtration, thiourea, HCl addition (reduction V-III)			0.0043	25.4		NA	
Sb(V) (+Sb(III))						0.0025	24.6			
As(III)	HG-AFS	Water	Filtration	l-cysteine, HCl	Tetrahydroborate on Amberlite IRA-400 resin	0.013	NA	19	3.8	[70]
As(V)						0.015	NA		3.6	
Total inorganic Fe	Spectrofluorimetry	Water, wine	NA	Pyoverdin immobilized on controlled pore glass (in flow cell), phthalate buffer, HCl	Persulphate immobilized on an ion-exchange resin	3	NA	NA	3.0–5.0	[71]
Inorganic Hg	Continuous-flow vapor generation AFS	Water, CRM (peach leaves)	Decomposition with HNO_3_, H_2_O_2_	SnCl_2_, thioglycolic acid (eluent)	Wool	0.01	NA	NA	NA	[72]
MeHg				SnCl_2_, thioglycolic acid (eluent), Br^−^/BrO_3_^−^						
Total dissolved Hg	AFS	Water	Filtration	HCl	Gold-coated SiO_2_	0.00018	2000	NA	NA	[73]
Total dissolved Hg	AFS	Water	NA	HCl	Active nano-structured gold	0.00008	10	9	1.1–3.1	[74]
total Hg	CV-AFS	Water	NA	BrCl, UV, HONH_2_·HCl, SnCl_2_,	Active gold	0.00004	NA	9	1.9	[75]
Hg	AFS	Water	filtration, acidification	HCl, UV, thermal desorption	Nano-gold collector	0.00008 *	NA	NA	3.7–5.6	[76]
Hg	UVG-AFS	Water	NA	DDTC, L-cysteine (eluent)	C_18_	0.00008	NA	9	˂5.0	[77]
CH_3_Hg^+^	AFS	Water, CRM (biological samples)	Filtration (water), KOH, methanol ultrasonic dissolving (CRM)	NA	Fe/SiO_2_/PDMS enrichment column, iron particle bed pyrolysis column	0.0002	108	NA	2.4	[78]
Hg(II)	CV-AFS	Water	Filtration, pH adjustment	HCl (eluent), NaBH_4_	IIP-Hg(HDz)_2_	0.02	29	5	5.2	[79]
Inorganic Hg	CV-AFS	Water	HCl, NaCl addition, photooxidation for total Hg determination	SnCl_2_, HCl	Hybrid ionic liquid-3D graphene-Ni foam	0.0036	180	2	4.1	[80]
Mn	FAFS	Water	Filtration, pH adjustment	HCl (eluent)	IDA chelating resin	0.9 nmol L^−1^	NA	22	2.9–4.8	[81]
Mn(II)	CL	Water	Filtration, pH adjustment	HCl (eluent), NaOH, Rh6G, diperiodatonickeleate (IV), Brij-35	8-HQ resin	0.5	NA	180	1.7–2.2	[82]
Pb	HG-AFS	Blood, hair, wine, urine, water	Digestion with HNO_3_ and HClO_4_ for blood, wine, and hair	HCl with potassium ferricyanide (eluent), NaBH_4_ in NaOH	Iminodiacetate chelating resin	0.004	11.3	50	1.6	[83]
Pb(II)	HG-AFS	Lead-free solder alloy	Digestion with HCl, HNO_3_, H_2_O_2_	Sodium citrate (eluent), HCl, KBH_4_ in KOH and K_3_Fe(CN)_6_	Macrocycle immobilized silica gel	0.003	61	40	1.8	[84]
Pb(II)	HG-AFS	Water, CRM (rice flour)	Filtration, pH adjustment, digestion with HNO_3_, H_2_O_2_ for CRM	HNO_3_ (eluent), KBH_4_	Multiwalled carbon nanotubes	0.0028	26	20	4.4	[85]
Se(IV)	HG-AFS	Water, CRM	Filtration, pH adjustment	APDC, HCl, KBH_4_ in NaOH	Polytetrafluoroethylene fiber	0.004	43	26	1.5	[86]
Se(IV)	HG-AFS	CRM (hair, milk powder, city waste incineration ash) urine, hair	Digestion with HNO_3_, HClO_4_, La^3+^ addition	NaOH, HCl, NaBH_4_	PTFE beads	0.005	11	38	1.2	[87]
Te(IV)	HG-AFS	Water, soil, sediment	Lixiviation with water (soil, sediment)	HCl, NaBH_4_	Fe_3_O_4_@ SiO_2_@NH_2_ (magnetic polymeric ionic liquid nanocomposite)	0.0019	67	48	4.3	[88]
Te(VI) (+Te(IV))			Heating with HCl (reduction Te(VI)), lixiviation with water (soil, sediment)			0.0037			5.1	
Knotted reactor										
As(III)	HG-AFS	Water, CRM	Filtration, acidification	HCl, APDC, KBH_4_	PTFE	0.023	11	32	1.3	[89]
Total inorganic As			Filtration, acidification, pre-reduction of As(V) to As(III) with l-cysteine						NA	
Hg(II)	CVG-AFS	Water, CRMs (water, rice flour, pork), fish muscle	Filtration, pH adjustment (water), homogenization, leaching (fish muscle)	DDPA, HCl, KBH_4_	PTFE	0.0036	13	30	2.2	[90]
CH_3_Hg				Dithizone, HCl, KBH_4_		0.002	24	20	2.8	
inorganic Hg	CV-AFS	Water	Acidification	DDTC, HNO_3_ (eluent) with electromagnetic induction heating, KBH_4_	PTFE	0.002	35	30	2.2	[91]
Hg(II)	In situ photochemical vapor generation AFS	CRMs (water, fish muscle, hair)	Sonication-assisted HCl leaching; dilution, pH adjustment (water)	DDTC, UV irradiation	PTFE	0.0008	NA	NA	4.5	[92]
CH_3_Hg^+^										
Se(IV)	HG-AFS	Water, CRMs (water, tea leaf)	Filtration, pH adjustment, spiking with La(NO_3_)_3_; drying, microwave digestion with HNO_3_, H_2_O_2_, spiking with La(NO_3_)_3_ (tea leaf)	Ammonium buffer, HCl (eluent), KBH_4_	PTFE	0.014	18	24	2.5	[93]

^1^ APDC: Ammonium pyrrolidinedithiocarbamate; CL: Chemiluminescence; CRM: Certified reference material; CV-AFS: Cold-vapor atomic fluorescence spectrometry; CVG-AFS: Chemical vapor generation atomic fluorescence spectrometry; DDPA: Diethyl dithiophosphate ammonium; FAFS: Flame atomic fluorescence spectrometry; HG-AFS: Hydride generation atomic fluorescence spectrometry; PTFE: Polytetrafluoroethylene; Rh6G: Rhodamine 6G; UV: Ultraviolet; UVG-AFS: Ultraviolet vapor generation atomic fluorescence spectrometry. ^2^ NA: Not available. * LOQ.

**Table 3:** Multisyringe FIA procedures for metal determination with fluorescence detection.

Analyte(s)	Analytical Technique ^1^	Sample	Pretreatment	Reagents	LOD, (µg L^−1^)	Sampling Frequency (h^−1^)	RSD (%)	Ref.
Acid-soluble As	HG-AFS	Soil	NA ^2^	Acetic acid extraction, K_2_S_2_O_8_ in NaOH, UV, thiourea with ascorbic acid, HCl, NaBH_4_	4	6 h	5.0–8.0	[105]
Reducible As				HONH_2_ *HCl extraction, K_2_S_2_O_8_ in NaOH, UV, thiourea with ascorbic acid, HCl, NaBH_4_	3.4			
Oxidizable As				H_2_O_2_ extraction, NaOH, UV, HONH_2_ *HCl, HCl, NaBH_4_	23.6			
As	HG-AFS	Paint, CRM (clay, water)	Microwave digestion with HNO_3_ and H_2_O_2_	KI, NaBH_4_, HCl	0.06	NA	≤6.2	[106]
Sb					0.03		≤6.6	
As	HG-AFS	Peanut, CRM (peach leaves)	Lyophilization, microwave digestion with HNO_3_ and H_2_O_2_, heating (reduction of Se(VI) to Se(IV))	HCl, NaBH_4_, KI with ascorbic acid	0.04	45	1.15–3.64	[107]
Sb					0.04		1.85–3.39	
Se					0.14		1.8–1.97	
Sb(III)	HG-AFS	Soil	Grounding, sieving, drying	Dowex 50W-X8 column, HCl, NaBH_4_	0.91 ng g^−1^	NA	3.2	[108]
Sb(V) + Sb(III)				KI, Dowex 50W-X8 column, HCl, NaBH_4_				
Total Sb				KI, HCl, NaBH_4_				
Selenite	HG-AFS	Recycling plant leachate, water	NA	HCl, NaBH_4_	0.11	33	2.56	[109]
Selenomethionine (+selenite)				UV, HCl, NaBH_4_	0.12	27	2.64	
Selenate (total Se)				KI, NaOH, UV, HCl, NaBH_4_	0.13	27	2.7	
Se(IV)	HG-AFS	Beer, CRM (dogfish muscle)	Pre-digestion with HNO_3_, ultrasound extraction, heating (inorganic Se)	KI, NaBH_4_, HCl	0.02	11	8.6	[110]
Total inorganic Se								
Se(IV)	CVG-AFS	Infusion tea	Heating tea in water at 80 °C	HCl, NaBH_4_	0.05	15	1.8–2.9	[27]
Total inorganic Se				KI, NaOH, UV, HCl, NaBH_4_				

^1^ CRM: Certified reference material; CVG-AFS: Chemical vapor generation atomic fluorescence spectrometry; HG-AFS: Hydride generation atomic fluorescence spectrometry; UV: Ultraviolet. ^2^ NA: Not available.

**Table 4:** Automated methods for metal determination that include chromatographic and electrophoretic separation techniques.

Analyte(s)	Analytical Technique ^1^	Sample	Sample Pretreatment	Reagents	Chromatographic Conditions	LOD (µg L^−1^)	RSD (%)	Ref.
Chromatographic techniques								
total As	HPLC–continuous flow–HG-AFS	CRM (soils)	Microwave-assisted phosphoric acid extraction	HCl, NaBH_4_	Anion-exchange column, ammonium phosphate buffers	0.02–0.04 mg kg^−1^	4.0–5.0	[122]
As(III), As(V), monomethylarsonate, dimethylarsinate	HPLC–continuous flow–HG-AFS	Plant CRMs, plant, soil	Grounding, microwave-assisted phosphoric acid extraction	NA ^2^	Anion-exchange column, ammonium phosphate buffers	5–8 ng g^−1^	6.0–10.0	[123]
As(III), As(V), monomethylarsonic, dimethylarsinic	SIA-HPLC-HG-AFS	CRM, seafood	Ultrasonic extraction with water	Standards, NaBH_4_, HCl	PRP-X100 column, phosphate buffers	0.023–1	NA	[124]
inorganic Hg, CH_3_Hg^+^, C_2_H_5_Hg^+^, C_6_H_5_Hg^+^	HPLC–microwave digestion–CV-AFS	Seafood	Extraction with CH_2_Cl_2_	K_2_S_2_O_8_ in HCl, KBH_4_	C_18_ column, methanol with tetrabutylammonium bromide and NaCl	0.14–0.3 ng	NA	[125]
CH_3_Hg^+^, C_2_H_5_Hg^+^, C_6_H_5_Hg^+^, Hg(II)	HPLC-CV-AFS	Seafood, CRM (dogfish muscle)	Homogenization, HCl leaching, neutralization	K_2_S_2_O_8_ in HCl, KBH_4_	C_18_ column, methanol, acetonitrile, water, APDC	4.75–6.75 ng g^−1^	1.7–2.9	[126]
CH_3_Hg^+^, C_2_H_5_Hg^+^, C_6_H_5_Hg^+^, Hg(II)	HPLC-CV-AFS	Fish	Cloud point extraction with Triton X-114 and APDC, HCl leaching	K_2_S_2_O_8_ in HCl, KBH_4_	C_18_ column, methanol, acetonitrile, water, acetic acid	0.002–0.009	1.2–3.4	[127]
CH_3_Hg^+^, C_2_H_5_Hg^+^, C_6_H_5_Hg^+^, Hg(II)	LC-CVG-AFS	CRM (dogfish muscle, hair)	Extraction with toluene, extraction with water	KBr/KBrO_3_ in HCl, NaBH_4_/N_2_H_4_	C_18_ column, cysteine in methanol, penicillamine in methanol, glutathione in methanol	16–20 pg	1.5–2.0	[128]
CH_3_Hg^+^, Hg(II)	HPIC-CV-AFS	CRM (dogfish liver, muscle, sediment)	Leaching with KBr, H_2_SO_4_, CuSO_4_, extraction with toluene, re-extraction with acidic thiourea	UV, H_2_O_2_, sodium ascorbate, SnCl_2_ in KOH	Thiol-functionalized silica resin column, thiourea, HCl, acetic acid	˂1 pg	NA	[129]
CH_3_Hg^+^, C_2_H_5_Hg^+^	GC-AFS	CRM, rice	Grounding, sieving, homogenization, extraction with CH_2_Cl_2_, re-extraction with Na_2_S_2_O_3_	NA	SPB–50 capillary column, N_2_	0.005 ng (as Hg)	1.3–2.5	[130]
CH_3_Hg^+^, C_2_H_5_Hg^+^, C_6_H_5_Hg^+^, Hg(II)	HPLC-UV-CVG-AFS	Seafood	Extraction with KOH, CH_2_Cl_2_, HCl, thiosulfate addition	UV, K_2_S_2_O_8_ in HCl, KBH_4_	Shim-pack CLC-ODS column, CH_3_CN, NH_4_Ac, 2-mercaptoethanol	0.2–1.01	NA	[131]
Hg(II), CH_3_Hg^+^	LC-CV-AFS	Biotic CRM	Leaching with acidic thiourea solution, KI addition	Polydivinylbenzene resin column, KBrO_3_, SnCl_2_, NaOH, Triton-X	Thiourea with acetic acid, anion–cation-exchange column	7, 4 pg g^−1^	NA	[132]
CH_3_Hg^+^, Hg(II)	HPLC-UV-CVG-AFS	Seafood	Digestion with KOH, CH_2_Cl_2_, HCl, l-cysteine addition	UV	Polymer-based-exchange column, acetonitrile, L-cysteine, pyridine, formic acid	0.08, 0.1	2.5–3.1	[133]
Hg(II), CH_3_Hg^+^, C_2_H_5_Hg^+^	HPLC-CV-AFS	CRM (dogfish muscle, lobster hepatopancreas)	Extraction with KOH, CH_2_Cl_2_, HCl	HCl, KBH_4_	Shim-pack CLC-ODS column, l-cysteine, ammonium acetate in water	0.05–0.1	1.4–2.5	[134]
Hg(II), CH_3_Hg^+^, C_2_H_5_Hg^+^	LC-CV-AFS	Hair	Leaching with acidic thiourea solution, KI addition	Polydivinylbenzene resin column, KBrO_3_, SnCl_2_, NaOH, Triton-X	Thiourea with acetic acid, anion–cation-exchange column	0.05–0.1 ng g^−1^	NA	[135]
CH_3_Hg^+^	Headspace trap GC-AFS	CRM (sediments)	Extraction with CuSO_4_, KBr, H_2_SO_4_, CH_2_Cl_2_, re-extraction into water, ethylation with NaBH_4_	NA	Polytetrafluoroethylene column, Ar	0.27 µg kg^−1^	3.1–3.7	[136]
CH_3_Hg^+^, Hg(II)	LC-UV-CV-AFS	Water	Filtration	2-mercaptoethanol, SnCl_2_	C_18_ column, APDC, methanol	0.015, 0.002	2–13	[137]
CH_3_Hg^+^	HPLC-CV-AFS	Water, CRM (hair)	Filtration, acidification (water); HCl leaching/extraction with TMAH (hair)	UV, BrO_3_^−^/Br^−^, HCl, SnCl_2_	Thiolsilica and thioureasilica column, APDC in methanol	0.00004 (as Hg)	NA	[138]
CH_3_Hg^+^	SPE-HPLC-CV-AFS	Rice	Grounding, microwave digestion with (CH_3_)_4_NOH	Br_2_, UV, SnCl_2_	Thiol/thiourea silica column, APDC in methanol, C_8_ column	0.12 ng g^−1^	NA	[139]
CH_3_Hg^+^, C_2_H_5_Hg^+^, Hg(II)	DLLME-HPLC-CV-AFS	Water	Filtration, pH adjustment	Acetone, 1-hexyl-3-methylimidazolium hexafluorophosphate, 2-mercaptoethanol, methanol, K_2_S_2_O_8_, KBH_4_, HCl	MP-C_18_ reversed-phase column, acetonitrile, ammonium acetate buffer, 2-mercaptoethanol	0.0015–0.003	NA	[140]
CH_3_Hg^+^, C_2_H_5_Hg^+^, Hg(II)	HPLC–barrier discharge plasma induced–CVG-AFS	CRM (tuna)	Digestion with KOH, CH_2_Cl_2_, HCl, sodium thiosulfate addition	NA	C_18_ column, 2-mercaptoethanol, methanol, NH_4_Ac	0.42–1.6	3.2–4.8	[141]
Sb(III), Sb(V), (CH_3_)_3_Sb^2+^	HPLC–pre-reduction–HG-AFS	Soil	Drying, oxalic acid extraction	l-cysteine, HCl, NaBH_4_	Anion-exchange column	0.07–1	4.5–5.1	[142]
Selenocysteine, selenomethionine, selenoethionine, Se(IV), Se(VI)	HPLC–microwave-assisted digestion–HG-AFS	Clam, prawn	Digestion with HNO_3_, HClO_4_, or enzymatic digestion with clean-up	KBrO_3_, HBr, NaBH_4_	C_18_ column, SAX column, potassium acetate	NA	NA	[143]
Se(IV), Se(VI), selenocystine, selenomethionine	LC-UV-HG-AFS	Water, oysters	Extraction with lipase, pronase, phosphate buffer	UV, KI, HCl, NaBH_4_	Anion-exchange column, phosphate buffer	19–60 pg	NA	[144]
Se(IV), Se(VI), selenocysteine, selenomethionine	LC-UV-HG-AFS	Milk	Enzymatic digestion	UV, HCl, heating, NaBH_4_	C_18_ column, tetraethylammonium chloride, water	0.4–1	0.8–3.2	[145]
Capillary electrophoresis								
**Analyte(s)**	**Analytical technique ^1^**	**Sample**	**Sample pretreatment**	**Reagents**	**CE conditions**	**LOD (** **µg L^−1^)**	**RSD (%)**	**Ref.**
CH_3_Hg^+^, C_2_H_5_Hg^+^, C_6_H_5_Hg^+^, Hg(II)	CE–volatile species generation–AFS	CRM (dogfish muscle)	Ultrasonic extraction with HCl solution and toluene, re-extraction with l-cysteine	HCl, KBH_4_	H_3_BO_3_, methanol (electrolyte), Pt electrode	6.8–16.5	1.8–6.3	[146]
Se(IV), Se(VI)	CE-HG-AFS	Water	Filtration	HCl, KBH_4_	NaH_2_PO_4_, cetyltrimethylammonium bromide (electrolyte), Pt electrode	25, 33	0.7–1.3	[147]

^1^ APDC: Ammonium pyrrolidinedithiocarbamate; CE: Capillary electrophoresis; CRM: Certified reference material; CV-AFS: Cold-vapor atomic fluorescence spectrometry; CVG-AFS: Chemical vapor generation atomic fluorescence spectrometry; DLLME: Dispersive liquid–liquid microextraction; GC: Gas chromatography; HG-AFS: Hydride generation atomic fluorescence spectrometry; HPIC: High-performance ion chromatography; HPLC: High-performance liquid chromatography; LC: Liquid chromatography; SIA: Sequential injection analysis; UV: Ultraviolet. ^2^ NA: Not available.

## Data Availability

Not applicable.

## Abbreviations

8-HQ	8-Hydroxyquinoline
CE	Capillary electrophoresis
CL	Chemiluminescence
CRM	Certified reference materials
CV-AFS	Cold-vapor atomic fluorescence spectrometry
CVG-AFS	Chemical vapor generation atomic fluorescence spectrometry
FAFS	Flame atomic fluorescence spectrometry
FIA	Flow-injection analysis
HG-AFS	Hydride generation atomic fluorescence spectrometry
HPEC	High-performance extraction chromatography
SIA	Sequential injection analysis
UVG-AFS	Ultraviolet vapor generation atomic fluorescence spectrometry
VSG	Volatile species generation
